# Resting-state functional magnetic resonance imaging-based identification of altered brain the fractional amplitude of low frequency fluctuation in adolescent major depressive disorder patients undergoing electroconvulsive therapy

**DOI:** 10.3389/fpsyt.2022.972968

**Published:** 2022-07-25

**Authors:** Xing-Yu Wang, Huan Tan, Xiao Li, Lin-Qi Dai, Zhi-Wei Zhang, Fa-Jin Lv, Ren-Qiang Yu

**Affiliations:** ^1^Department of Radiology, The First Affiliated Hospital of Chongqing Medical University, Chongqing, China; ^2^Department of Psychiatry, The First Affiliated Hospital of Chongqing Medical University, Chongqing, China

**Keywords:** major depressive disorder (MDD), adolescent, fALFF, electroconvulsive therapy, resting-state fMRI

## Abstract

**Purpose:**

While electroconvulsive therapy (ECT) has been repeatedly been shown to effectively and efficiently treat the major depressive disorder (MDD), the mechanistic basis for such therapeutic efficacy remains to be firmly established. As such, further research exploring the ECT-based treatment of MDD in an adolescent population is warranted.

**Methods:**

This study included 30 treatment-naïve first-episode MDD patients and 30 healthy control (HC) individuals (aged 12–17 years). All participants were scanned using rs-fMRI, and the 30 MDD patients were scanned again after 2 weeks of the ECT treatment period. Intrinsic local activity in each voxel was assessed based on the fractional amplitude of low frequency fluctuation (fALFF) parameter, with all fALFF analyses being completed using the REST application. Correlations between ECT-related changes in fALFF and clinical parameters were additionally examined.

**Results:**

Relative to HCs, MDD patients exhibited increased fALFF values in the right inferior frontal gyrus (ORBinf), inferior occipital gyrus (IOG), and the left middle frontal gyrus (MFG) at baseline. Following ECT, these patients exhibited significant increases in fALFF values in the right medial superior frontal gyrus (SFGmed), dorsolateral superior frontal gyrus (SFGdor), anterior cingulate, and paracingulate gyrus (ACG), median cingulate and paracingulate gyrus (DCG), and left MFG. MDD patient HAMD scores were negatively correlated with fALFF values when analyzing pre-ECT vs. post-HCT ΔHAMD and fALFF values in the right SFGmed, SFGdor, and the left MFG.

**Conclusion:**

These data suggest that ECT induced altered fALFF in some regions of the brain, suggesting that these alterations may serve as a neurobiological indicator of ECT effectiveness in MDD adolescents.

## Introduction

Major depressive disorder (MDD) is a potentially life-threatening psychiatric condition that occurs in response to a range of genetic factors, environmental inputs, stressors, and other factor (1–3), resulting in symptoms including persistent depression, cognitive impairments, and high levels of morbidity including an elevated risk of suicide, with MDD patients exhibiting a lifetime suicide rate of 2–12% (4). In recent years, the number of individuals diagnosed with MDD has risen substantially, particularly among younger segments of the population, with adolescents in particular being highly susceptible to depression (5). Some estimates suggest suicide to be the second most common cause of death for persons 10–24 years of age (6). Owing to the marked structural changes in the brain that occur during adolescence, it is regarded as an important window of susceptibility for the onset of MDD (7, 8). Lee et al. (9) noted that the drivers of depression are highly diverse and complex, with adults and adolescents diagnosed with MDD exhibiting distinct clinical. In some cases, adolescents suffering from depression are more likely to develop difficult-to-treat conditions such bipolar disorder, comorbid borderline personality disorder, suicidal thoughts, and self-harming behaviors (10, 11).

Electroconvulsive therapy (ECT) (12, 13) is an approach wherein a brief jolt of electricity is administered to the brain in an effort to treat certain mental disorders, resulting in the simultaneous firing of cells throughout the brain, resulting in convulsions and altered neural metabolic processes that can improve MDD patient outcomes (14). Several studies have explored the drivers of abnormal brain activity and ECT-related therapeutic benefits. For example, Liu et al. (15) conducted a systematic analysis of the effects of ECT-related antidepressant activity and reported a correlation between such activity and subgenual anterior cingulate activity and connectivity in MDD. Moreover, Strober et al. (16) explored the impact of ECT on adolescents diagnosed with severe endogenous depression such that they were able to identify certain phenomenological characteristics predictive of ECT responses. Consoli et al. (17) assessed the effects of ECT on severe forms of treatment-resistant self-injurious behavior and aggression (SIB/AGG) in young individuals diagnosed with intellectual disabilities and comorbid psychiatric disorders, confirming the efficacy of ECT in this setting. As such, these results suggest a close link between the mechanism whereby ECT exerts its therapeutic activity and neurological activity in humans, although further work is essential to fully elucidate these mechanisms in adolescent MDD patients.

Resting-state functional magnetic resonance imaging (rs-fMRI) is a high-performance imaging modality that is easy to operate and resistant to interference (5, 18), leading to its widespread use in the analysis of brain function, neural network activity, and in the diagnosis of a range of conditions impacting the central nervous system (19, 20, 21). In addition, rs-fMRI can be leveraged to study the pathogenesis of MDD and treatment-related changes in brain activity. Of the parameters that can be measured in this neuroimaging context, regional cerebral blood flow (rCBF), blood oxygen level-dependent (BOLD) signal, and deoxyhemoglobin content (22) values attributable to low-frequency unconscious brain activity are used to assess resting-state amplitude of low-frequency fluctuation (ALFF) (23) values, providing an accurate and highly representative means of examining brain activity based on fMRI data (24, 25).

As an improved ALFF method (26, 27), the fractional amplitude of low frequency fluctuation (fALFF) can eliminate the impact of physiological noise on the resultant data while providing greater specificity and sensitivity for the detection of spontaneous brain activity based on the use of average signal oscillation intensity in the 0.01–0.08 Hz range and the ratio of the whole frequency band oscillations.

Here, rs-fMRI was used to explore the mechanisms underlying the efficacy of ECT in adolescent patients diagnosed with MDD. Specifically, fALFF data pertaining to these MDD patients’ brain activity was compared before and after ECT treatment through an fMRI approach, enabling us to explore the processes underlying the ECT’s antidepressant effects in this patient group.

## Materials and methods

### Participants

This study enrolled 30 MDD patients and 30 healthy control (HC) individuals 12–17 years of age. All patients with MDD in this study were recruited from inpatient clinics at the Department of Psychiatry of the First Affiliated Hospital of Chongqing Medical University, China, from October 2019 to October 2021. Patients were eligible for study inclusion if they were experiencing first-episode depression without any prior history of mania or hypomania, had no history of antidepressant treatment, were right-handed, were of Han ethnicity, exhibited a Hamilton Depression Scale (HAMD-24) score > 17, were 12–17 years of age, had not taken any psychotropic drugs, and had not used sedative, anesthetic, or analgesic drugs within the past 1-month period. Patients were excluded from this study if they had any prior history of mental health disorders including bipolar disorder or schizophrenia, had been diagnosed with organic brain diseases or other serious physical illnesses, reported substance abuse or dependence, or exhibited contraindications for MRI scanning. Patients with MRI scan contraindications.

HC study participants were recruited from as volunteers from local schools, and were eligible for inclusion if they exhibited a HAMD-24 score < 7, were right-handed, were of Han ethnicity, were 12–17 years of age, and weren’t suffering from any severe mental or physical illness. Exclusion criteria for HCs were identical to those for MDD patients.

Adolescents and their parents or guardians gave their written informed permission to participate in the research, and the Human Research and Ethics Committee at the First Affiliated Hospital of Chongqing Medical University gave its approval (no.2017-157).

### Electroconvulsive therapy

A Thymatron DGx instrument (Somatics, LLC., Venice, IL, United States) was used to perform modified bi-frontotemporal ECT for all patients at the First Affiliated Hospital of Chongqing Medical University. The initial three ECT courses were performed on consecutive days, with the remaining courses then being completed every other day. No courses were performed on weekends. After a 2-week treatment course, the ECT process was complete. The energy level used for ECT was determined based on the age of the patient being treated (energy percentage = age × 0.5%), with stimulation energy then being adjusted based on seizure time, increasing by 5% during subsequent treatments when the seizure time was < 25 s. Succinylcholine (0.5–1 mg/kg) and diprivan (1.5–2 mg/kg) were used to induce anesthesia. In this study, individuals were treated with antidepressants.

### Resting-state functional magnetic resonance imaging data acquisition

A 3T GE Signa HDxt scanner (GE Healthcare, Boston, IL, United States) equipped with an 8-channel head coil was used for rs-fMRI scanning of all participants. During scanning, participants were directed to remain still and awake with their eyes closed while not thinking about anything specific to the greatest extent possible. Head motion and machine noise were, respectively, mitigated using foam pads and earplugs. All echoplanar imaging pulse sequences were performed with the following parameters: repetition time (TR) = 2000 ms, echo time (TE) = 40 ms, field of view (FOV) = 240 mm × 240 mm, matrix = 64 × 64, flip angle = 90^°^, slice number = 33, slice thickness/gap = 4.0/0 mm; scanner time = 8 min, and 240 volumes. Three-dimensional T1-weighted MRI scans used for rs-fMRI co-registration were performed using the following settings: TR = 24 ms; TE = 9 ms; FOV = 240 mm × 240 mm, matrix = 256 × 256, flip angle = 90^°^, and slice thickness/gap = 1.0/0 mm.

### Data preprocessing

The SPM software platform and the DPABI tool^1^ (28) were used to process fMRI data. The initial 10 images for each patient were discarded to ensure sufficient time for adaptation to the scanning process, with the remaining 230 volumes then being analyzed. Data were corrected for head movement, and patients were not eligible for inclusion in subsequent analyses If they exhibited a maximum displacement of > 1.5 mm along the x, y, or z axes or angular displacement > 1.5°. Slice correction was performed to control for acquisition delay. All fMRI images were subjected to normalization and registered to the standard Montreal Neurological Institute (MNI) with 3 mm× 3 mm × 3 mm resampling. Images were also smoothed with an 8-mm full-width at half-maximum Gaussian kernel and were bandpass filtered (0.01–0.08Hz) to eliminate low-frequency drift and high-frequency noises including respiratory and cardiac noise.

### The fractional amplitude of low frequency fluctuation calculations

The REST software was used to measure fALFF values, which served as a measure of the intrinsic local spontaneous neuronal activity in each voxel. For these analyses, individual processed rs-fMRI datasets were transformed via fast Fourier transformation to a frequency domain, followed by the calculation of the square root of the power spectrum. Then, fALFF values were established based on the averaged square root across the 0.01–0.1 Hz range. Then, to account for local variations, we normalized each participant’s voxels levels fALFF values by their global mean fALFF value. Overall, this approach allowed fALFF to be determined as the mean power spectrum in a specific low-frequency band (0.01–0.1 Hz) by dividing the spectrum in that band by the spectrum throughout the entire frequency range (29).

### Statistical analyses

Differences in patient clinical and demographic characteristics before and after treatment, continuous variables were compared using paired *t*-tests. Paired sample *t*-tests were used to examine changes in fALFF before and after ECT therapy in SPM12, and SPSS v26.0 (IBM, Chicago, NY, United States) was used for all other analyses with a false discovery rate (FDR)-corrected *P* value < 0.05 as the cutoff for significance. Significant variations in patient clinical symptoms, as defined by HAMD scores, were correlated with mean values for specified parameters in various brain areas using Pearson correlation analysis. Similarly, Pearson correlation analyses were used to determine whether there was a statistically significant relationship between fALFF values and the degree to which clinical symptom changes (ΔHAMD = pre-ECT HAMD – post-ECT HAMD).

## Results

Table 1 lists the demographic characteristics of the participants. At baseline, no significant differences in age, sex, or level of education were found between MDD patients and HCs (*P* > 0.05). Following a 2-week ECT treatment period, MDD patients exhibited significant reductions in total HAMD scores (*P* < 0.05) (Table 2).

**TABLE 1:** Demographics and clinical characters of healthy controls (HCs) and MDD patients.

Demographic data	MDD (*n* = 30)	HCs (*n* = 30)	*T* (orx^2^)	*P*-value
Gender (male/female)	30(9/21)	30(8/22)	0.774	0.082*^a^*
Age (years)	14.77 ± 1.43	15.50 ± 1.87	−1.705	0.093*^b^*
Years of education (years)	8.90 ± 1.729	9.83 ± 2.198	−1.828	0.073*^b^*
HAMD score	28.63 ± 6.01	0.10 ± 0.403	25.952	0.001

^a^The *p* value for gender distribution was obtained by chi-square test.

^b^The *p* value were obtained by two sample I-tests.

NC, normal control; HAMD, Hamilton depression scale.

**TABLE 2:** Comparisons of the HAMD scoress between pre- and post-ECT.

	pre-ECT	post-ECT	T-value	*P*-value
HAMD score	28.63 ± 6.01	13.70 ± 8.929	7.600	<0.001

**FIGURE 1:**
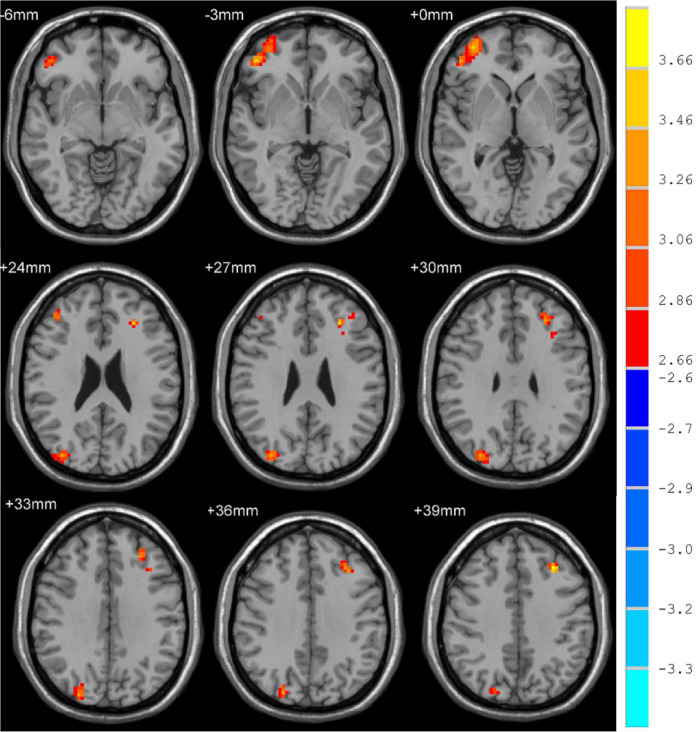
Brain regions where fALFF levels are significantly different in MDD patients to that of healthy controls.

Whole-brain voxel-level analyses revealed significant increases in fALFF in the right orbital inferior frontal gyrus (ORBinf), inferior occipital gyrus (IOG), and left middle frontal gyrus (MFG) in MDD patients relative to HCs (Figure 1) when using a cluster threshold of *P* < 0.05 (*P* < 0.001, Voxel size > 50). However, for these areas, we found no statistically significant associations between HAMD scores and fALFF values that exhibited significant baseline differences between individuals with MDD and controls.

Subsequently, MDD patients exhibited significant alterations in whole-brain fALFF. Specifically, fALFF was significantly elevated in the right medial superior frontal gyrus (SFGmed), anterior cingulate and paracingulate gyrus (ACG), median cingulate and paracingulate gyrus (DCG) dorsolateral superior frontal gyrus (SFGdor) and the left middle frontal gyrus (MFG) following treatment with a cluster threshold of *P* < 0.05 (*P* < 0.001, Voxel size > 50).

**FIGURE 2:**
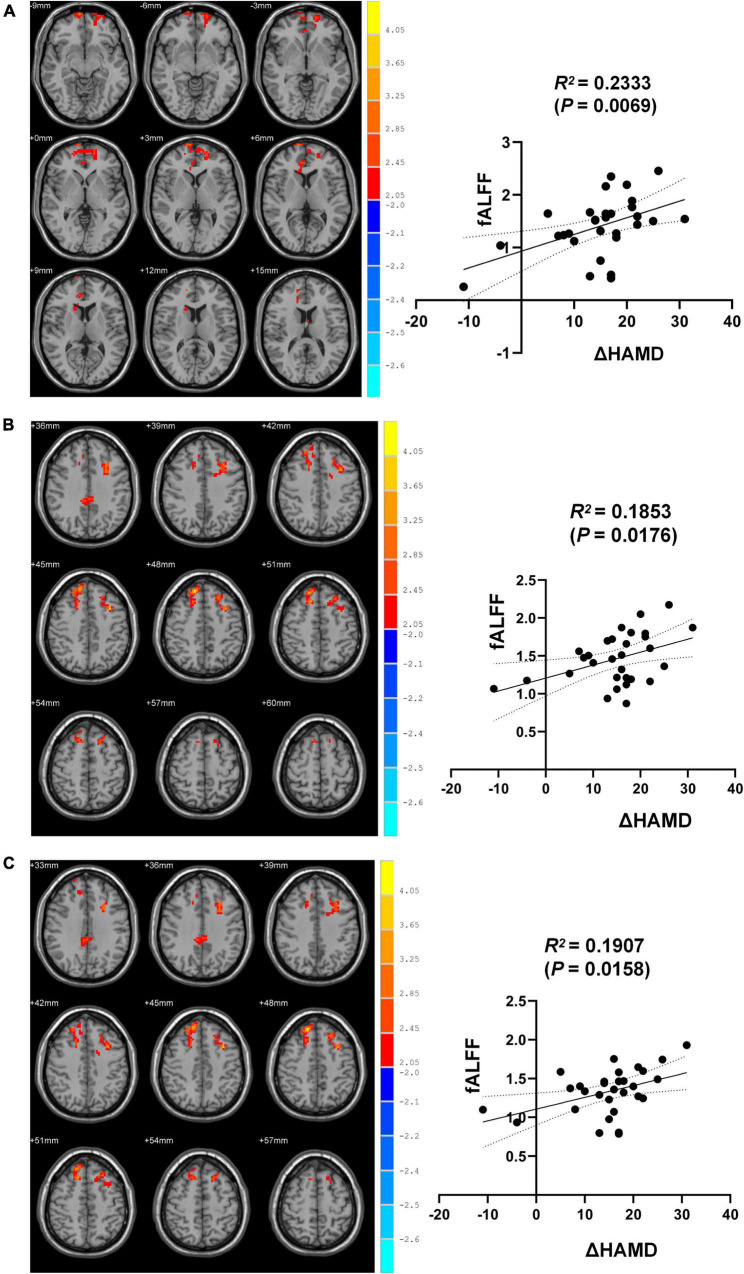
Brain regions differences between pre-and post-ECT. **(A)** Correlation analysis of fALFF in brain regions (the right SFGmed) with difference and ΔHAMD. **(B)** Correlation analysis of fALFF in brain regions (the right SFGdor) with difference and ΔHAMD. **(C)** Correlation analysis of fALFF in brain regions (the left MFG) with difference and ΔHAMD.

Correlation analyses revealed ΔHAMD to be significantly negatively correlated with fALFF in the right SFGmed, SFGdor, and left MFG when comparing pre- and post-ECT data (*R*^2^ = 0.2333, *P* = 0.0069; *R*^2^ = 0.1853, *P* = 0.0176; *R*^2^ = 0.1907, *P* = 0.0158) (Figure 2).

## Discussion

All MDD patients enrolled in the present study were treatment-naïve at the time of enrollment and exhibited HAMD-17 scores that were significantly lower following ECT treatment consistent with the improvement of depressive symptoms. Following ECT, MDD patients exhibited significant increases in fALFF in the right SFGmed, ACG, SFGdor, DCG, and left MFG. A negative correlation was observed between ECT effectiveness and fALFF changes in the right SFGmed, SFGdor, and left MFG based on correlation studies. Several studies of MDD to date have identified a direct link between the incidence of depressive symptoms and reductions in the levels of certain excitatory neurotransmitters in the prefrontal lobe with corresponding decreases in frontal lobe neuronal synapses (30–32), with this region being one of the most commonly impaired in MDD patients (32). Overall, fALFF values in the SFGmed.R, SFGdor.R and MFG.L regions were significantly increased following ECT.

Prior work has shown MDD patients to exhibit changes in ALFF/fALFF in numerous brain regions such as the right SFGmed, ACG, SFGdor, DCG, and left MFG. Individuals with MDD and those at a greater risk for developing MDD have been shown to have frontal lobe abnormalities, both anatomical and functional, these results have yet to be leveraged to effectively treat or prevent this debilitating psychiatric condition. Hongqing et al. (33) employed an MRI approach to examine potential drivers of suicidal ideation among adolescents suffering from depression, ultimately determining that these MDD patients exhibited abnormal levels of autonomic neural activity in the left hippocampus and left MFG. The left MFG is a component of the left dorsolateral prefrontal cortex (DLPFC), which primarily governs executive function and cognition (34), in addition to being linked to processes including working memory (35), social perceptions and the processing of social information (36), memory retrieval (37), emotional regulation, and processing emotional stimuli (38). Li et al. (39) determined through a comparison of non-remitting and remitting patients that the former group exhibited reductions in gray matter volume in the left DLPFC, potentially suggesting that MDD patients with remittent disease may exhibit distinct morphological and cognitive features from patients with non-remittent disease. Accordingly, voxel-based structural changes in this region may be characteristic of a subset of recurrent MDD patients that fail to reliably respond to antidepressant treatment. Kong et al. additionally reported increased ALFF values in the left MFG, right MFG, and orbital regions in line with the results of the present analysis, although they also determined that elderly MDD patients exhibited reductions in ALFF values in the right SFG and MFG following ECT (40), potentially suggesting the existence of marked differences between adolescent and elderly MDD patients, in line with other data from a study performed by Lee et al. (9).

To date, few analyses have specifically focused on ECT-related changes in fALFF values in the right SFG, including the right SFGmed, and SFGdor, in adolescent MDD patients. Most studies that have explored ECT-related changes in adolescent MDD patients have reported altered ALFF/fALFF values in the precentral gyrus, right fusiform gyrus, left MFG, and right middle temporal pole gyrus. Even so, studies focused on various other psychiatric conditions have demonstrated a relationship between the right SFGmed and SFGdor and treatment outcomes (41–44).

Wang et al. (45) reported gray matter volume in the right SFG to be significantly positively correlated with superiority bias, suggesting a structural basis for such bias. Li et al. (39), in contrast, found that reductions in gray matter volume in the right SFG were not sufficient to differentiate between MDD patients that did and did not achieve remission. Regional homogeneity (ReHo) values in the right SFG and superior MGF in bipolar disorder patients have previously been found to be higher than those for MDD patients, suggesting that these values can be leveraged to distinguish between patients with these two psychiatric disorders (46). Prerona et al. (47) employed an rs-fMRI approach to analyze schizophrenia patients, and found that elevated activity in the right MFG and SFG was positively correlated with elevated levels of consciousness or subliminal activity. Han et al. (48) additionally found the right SFG and MFG to be closely linked to a range of psychiatric disorders, suggesting that fALFF intensity may correspond to a neurological mechanism tied to the treatment of certain mental health conditions.

Rosalux et al. (41) established a close relationship between the right SFG and the reappraisal of the emotional impact of particular events, with this ability and associated activity being linked to long-term mental health outcomes. Li et al. (43) additionally found fALFF levels in the right precuneus and SFGdor to be significantly negatively correlated with extraverted behavior among adolescents and children, suggesting a role for the right SFGdor in the regulation of emotion. By combining imaging and genetic strategies, Yuan et al. (44) further explored biomarkers linked to the diagnosis of MDD and to the prediction of therapeutic outcomes, ultimately establishing that relative to non-responding depression, responsive depression exhibited lower right SFGdor nodes, with the ALFF values in the bilateral occipital gyrus (MOG), left lentiform nuclear, right superior temporal gyrus, and the CBF in the right calcarine gyrus, and left caudate nucleus being significantly correlated with baseline MDD severity or early efficacy. In their study, analyses of the CBF of the left caudate and the right MFG together with the ALFF of the right inferior temporal gyrus were better able to predict non-responsive depression. Cui et al. (49) further performed the rs-fMRI-based experimental evaluation of brain functional changes in idiopathic trigeminal neuralgia patients, ultimately establishing a link between increased fALFF and sensory integration or pain regulation in these patients.

The left MFG is involved in an important role in emotional regulation, executive function, cognition, working memory, and the processing of emotional stimuli. Right MFG activity levels are positively correlated with the strength of consciousness or subliminal activities (47). The right SFG is also associated with the reappraisal of the emotional impact of particular events and associated long-term mental health outcomes (41). Here, MDD patients were found to exhibit significant increases in the fALFF of the right medial SFG, the right ACG, the right SFGdor, the left MFG, and the right median DCG. Through correlation analyses, the efficacy of ECT treatment was found to be negatively correlated with changes in neuroimaging findings in the right SFGmed, SFGdor, and left MFG regions when comparing pre- and post-treatment fMRI images. This may thus support a model wherein the therapeutic effects of ECT are dependent on the synergistic treatment of these three regions of the brain. Brain networks that facilitate systemic adaptation and flexibility are responsible for small-worldness (42) which is defined as the dynamic remodeling of the small-world topology and related community structure. Functional connectivity between the right precuneus and the right SFGdor was discovered by Li et al. (43) to mitigate the mediating effects of small-worldness. Similarly, these findings are consistent with the hypothesis that the right SFGmed, SFGdor, and the left MFG all have a role in determining patient outcomes via processes analogous to small-worldness in MDD.

Overall, the results of these prior studies are only partially consistent with the results of the present analyses. These inconsistencies may be attributable to differences in the therapeutic approaches, diagnoses, disease severity, and patient assessment methods employed in these various research efforts. In addition, differentiating between unipolar and bipolar depression in adolescent individuals can be challenging, complicating these research efforts. Few studies used fMRI-based studies to determine ECT efficacy in treating MDD in adolescents. The study findings may suggest the mechanism by which ECT improves outcomes for MDD patients by pointing to changes in fALFF across many brain regions.

## Conclusion

In summary, the results of this study revealed that ECT was able to effectively treat MDD in adolescents while offering direct insight into ECT-related neuroimaging changes through fMRI analyses. Specifically, following ECT, MDD patients exhibited significantly increased fALFF in the right SFGmed, ACG, SFGdor, DCG, and left MFG. Correlation analyses revealed ECT clinical efficacy (ΔHAMD) to be significantly negatively correlated with changes in fALFF in the right SFGdor, SFGmed, and in the left MFG when comparing pre- and post-ECT results. As such, these data suggest a potential therapeutic mechanism whereby ECT can synergistically alter brain activity in the right SFGmed, SFGdor, and left MFG.

## Limitations

This study is subject to certain limitations. Firstly, this study had a small sample size. Furthermore, it is not feasible to rule out the possibility that these findings were influenced by resting respiratory and cardiac rhythms. Moreover, all patients underwent antidepressant therapy during the course of ECT treatment, thus potentially impacting brain function.

## Data availability statement

The original contributions presented in this study are included in the article/**Supplementary material**, further inquiries can be directed to the corresponding authors.

## Ethics statement

The studies involving human participants were reviewed and approved by the Human Research and Ethics Committee of The First Affiliated Hospital of Chongqing Medical University. Written informed consent to participate in this study was provided by the participants’ legal guardian/next of kin. Written informed consent was obtained from the individual(s), and minor(s)’ legal guardian/next of kin, for the publication of any potentially identifiable images or data included in this article.

## Author contributions

X-YW and HT: writing – original draft and scanning MRI data. HT: analyzed the data. XL and L-QD: investigation. Z-WZ, F-JL, and R-QY: conceptualization, checking the data, methodology, and writing – review and editing. All authors contributed to the article and approved the submitted version.

## Footnotes

1
http://www.restfmri.net
